# Recreation

**Published:** 1882-01

**Authors:** 


### RECREATION.

THE literal meaning of this word is to make over again; but in its ordinary acceptation it is intended to convey the idea of rest, refreshment, or rather, renovation. The body is refreshed by rest; the brain is renovated by sleep, by absolute repose. But both body and brain may be invigorated for a season, by changing the direction of their respective activities; and also by working alternately. A man who has become tired of riding on horseback or in a carriage, rests himself, gets rid of his fatigue, by walking. The brain which has become weary in thinking of one subject is refreshed by taking up some other study. On the other hand, a man who feels tired all over, by work, or a long walk, will "get rested" sooner by sitting down to read than if he did nothing. Rachel, the great tragic actress, when returning from one of her performances at two or three o'clock in the morning, rested herself by spending an hour or two in changing the furniture of her rooms. The best sedative which a public speaker can take after a great effort, is to read a newspaper, or any thing else which has a variety of short statements.

The great practical idea we wisn to convey is, that recreation is not idleness, but a change of direction in the operation of the physical or mental forces. A French actress lately went mad within an hour after the play, because she went home, laid down and let the mind run on in the same track. She should have changed to bodily activity, like Rachel.

**A NATURAL WISH.**—It is very natural that we should wish the circulation of this JOURNAL largely extended. Has it ever occurred to our subscribers that it is their duty to do something toward this extension? If you have derived benefit from it, would it not be a kindly and neighborly act to induce your nearest friends to take it a year on trial? for it is very certain that by following its counsels, their health and happiness would be promoted and their lives extended for years.

HAVE YOU CHILDREN? You know how indifferent they are to your advice in too many cases; you know also that more importance is attached to what they see in print than to what you might say in regard to health. Would not the chances of their being benefited be greatly increased if you were to order the JOURNAL for each one of them? Their illness would be your greatest trouble, their death an irremediable grief; and yet thousands of lives are lost every year from inattention to the suggestions of one single article, to wit—about cooling off slowly after exercise.

